# Dynamic Nature of Soil Iron Minerals: Recent Advances Using Iron Isotope Tracers and Mössbauer Spectroscopy

**DOI:** 10.1021/acs.est.6c07339

**Published:** 2026-10-15

**Authors:** Luiza Notini, Andrew R. C. Grigg, L. Joëlle Kubeneck, Michelle M. Scherer, Ruben Kretzschmar

**Affiliations:** † Department of Civil, Construction, and Environmental Engineering, 5972 University of Delaware, Newark, Delaware 19716, United States; ‡ Institute of Biogeochemistry and Pollutant Dynamics, Department of Environmental Systems Science, 27219 ETH Zurich, CHN, 8092 Zurich, Switzerland; § Faculty of Science and Engineering, 4571 Southern Cross University, Military Road, East Lismore 2860, Australia; ∥ Department of Microbiology, Radboud Institute for Biological and Environmental Sciences, Radboud University, 6525 AJ Nijmegen, The Netherlands; ⊥ Geological Survey of the Netherlands, Netherlands Organization of Applied Scientific Research (TNO), Princetonlaan 6, Utrecht 3508 TA, The Netherlands; # Civil, Environmental, and Geospatial Engineering, Michigan Technological University, Houghton, Michigan 49931, United States

**Keywords:** ^57^Fe isotope tracers, Fe redox cycling, soil and sediment biogeochemistry, atom exchange, reductive dissolution, soil incubations, mineral-slurry studies

## Abstract

Iron plays a central role in defining the redox and surface chemical environment of soils and sediments, by mediating the behavior of nutrients, pollutants, and carbon compounds. However, the mechanisms that drive dynamic changes in Fe speciation and mineral composition can be difficult to trace. Over three decades, technical advances in spectroscopy and theoretical modeling techniques have revealed detailed mechanisms of Fe mineral recrystallization and transformation. Here, we provide a perspective on the application of iron isotopic tracers to the development of atomic-scale mechanistic understanding of Fe mineral dynamics. We review key milestones in the advancement of ^57^Fe tracers coupled with ^57^Fe Mössbauer spectroscopy and draw links between the understanding of Fe dynamics with ^57^Fe tracers and biogeochemical cycling of other elements in soils and sediments. Iron isotopes are increasingly used in Fe biogeochemistry research, and our analysis will point out unique opportunities offered by ^57^Fe tracers to unravel important questions in soil biogeochemistry today.

## Graphical abstract

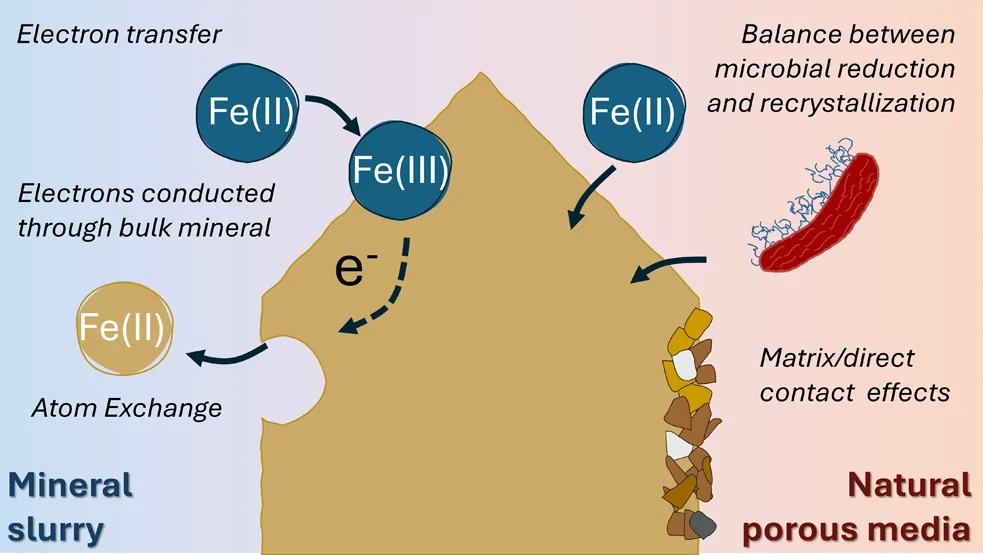

## Introduction

Iron (Fe) is the fourth most abundant element in the Earth’s crust and a primary constituent of most soils and sediments. During mineral weathering, Fe precipitates into a diverse suite of secondary minerals, including oxides, oxyhydroxides, phosphates, carbonates, and sulfides.^1^ All of these secondary minerals possess reactive surfaces controlling nutrient and contaminant mobility.^1^ Furthermore, in suboxic to anoxic environments, Fe serves as a major terminal electron acceptor in microbial respiration and the Fe­(II)–Fe­(III) redox couple acts as a critical metabolic bridge.^2,3^

Biogeochemical redox reactions involving Fe minerals are fundamental to all redox-active environmental systems, from wetlands and estuaries to aquifers and marine sediments. Beyond their role as passive sorbents, Fe minerals function as electron shuttles and catalysts for surface reactions involving both inorganic and organic compounds. Therefore, Fe mineral transformations are of interest in a variety of fundamental and applied scientific fields ranging from soil and aquatic environmental sciences, wastewater treatment, archeology, to planetary sciences.^4–8^

Over the past several decades, research has demonstrated that microbially driven redox cycling can result in the complete recrystallization or structural transformation of Fe minerals.^9,10^ However, the stability and reactivity of Fe minerals reflect interactions between mineral surfaces and their chemical environment. This recognition dates back to the seminal observations of Schwertmann, who observed that the presence of organic matter in a natural ferrihydrite prevented its transformation to goethite under conditions where pure ferrihydrite readily crystallizes to goethite within days.^11^ These findings laid the foundation for the now well-established role of carbon in stabilizing poorly crystalline Fe (oxyhydr)­oxides^12,13^ which in turn, suppresses its own availability for microbial mineralization, thereby shaping soil fertility and climate dynamics.^13–16^

Together, these insights have fundamentally shifted our understanding of Fe biogeochemistry. Field, lab, and theoretical work show that the mineral–water interface hosts molecular-scale processes regulating the global cycling of carbon, nutrients, and trace metals. Yet tracking Fe mineral transformations in complex environmental matrices has long been challenging. Traditional mineralogical techniques, such as X-ray diffraction, are unable to quantify amorphous, short-range-ordered, and minor crystalline Fe phases in soils, and sequential extractions are not mineral-specific, yielding only operationally defined pools that typically lack the precision needed to trace atomic-scale Fe mineral transformation pathways. Even element-specific techniques, including synchrotron-based spectroscopy, can struggle to quantify Fe phases embedded in soil matrices.

To overcome this analytical challenge, we can take advantage of iron’s four stable isotopes (percent natural abundance): ^54^Fe (5.84%), ^56^Fe (91.76%), ^57^Fe (2.12%), and ^58^Fe (0.28%).^17^ Of these, only ^57^Fe is Mössbauer active, making ^57^Fe Mössbauer spectroscopy (hereafter referred to as MBS) an element and isotope-specific spectroscopy. MBS quantifies Fe-oxidation states, mineral phases, magnetic ordering, and crystallinity. A recent paper by Thompson et al.^18^ provides detailed protocols, practical considerations, and limitations for its application in soil and sediment research. Despite some challenges in spectral interpretation in complex environmental samples, MBS, combined with other techniques, has been essential for characterizing Fe minerals in soils and sediments.^18^ However, despite its strengths, MBS alone offers a limited ability to directly trace dynamic transformation pathways.

Here, we highlight how the combined use of stable Fe isotope tracers and MBS can overcome this limitation. By selectively probing ^57^Fe atoms, while other Fe isotopes remain invisible, this approach enables tracking of Fe during redox transformations even in complex environmental samples. Figure 1 illustrates different ways of using ^57^Fe to trace Fe atom dynamics in electron transfer and atom exchange experiments. Fe tracers are initially added in the aqueous phase (Figure 1A),^19^ in the solid phase (Figure 1B),^20,21^ in sequential aqueous Fe^2+^ spikes (Figure 1C),^22^ or in one Fe phase in mixtures with other minerals (Figure 1D).^23,24^ Extending the application to soils and sediments, the use of ^57^Fe and MBS allows tracing of atoms in the aqueous phase (Figure 1E),^25,26^ of specific mineral mixed into a complex matrix (Figure 1F),^27^ and of the in situ transformation of the synthesized mineral (Figure 1G).^28–30^ These experimental “isotope games” have advanced our understanding of mineral transformations at the atomic scale, and provided insights into mineral reactivity in natural environments.

**Figure 1:**
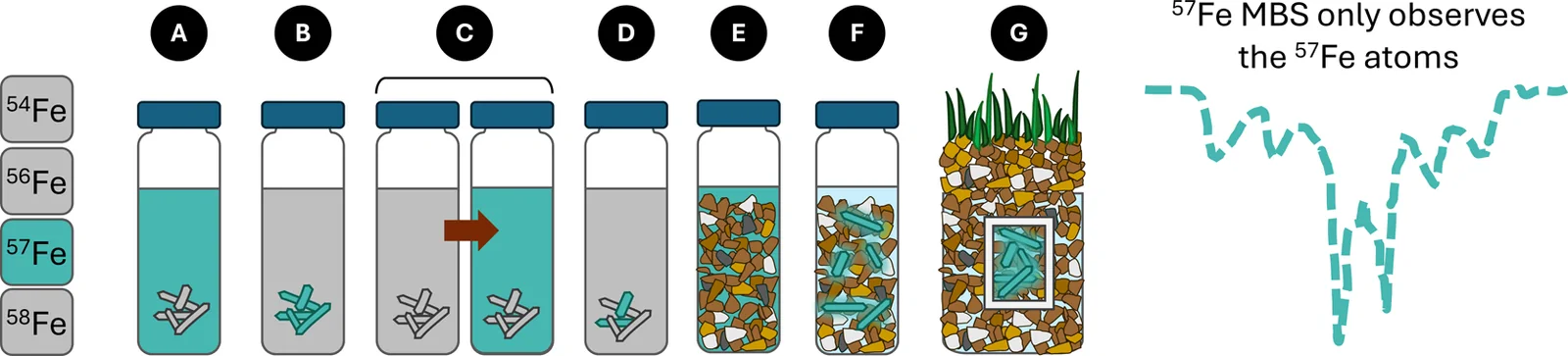
Illustration of different ways of using ^57^Fe as isotope tracer (marked in cyan) in systems containing predominantly MBS-invisible Fe isotopes (marked in gray), followed by ^57^Fe detection by Mössbauer spectroscopy. Illustrations A to D focus on approaches to differentiate the Fe pools in controlled laboratory experiments, while approaches E to G leverage the ^57^Fe isotope to observe the fate of the added Fe atoms in systems that contain native Fe atoms.

### Spectroscopic Proof of Electron Transfer and Fe Atom Exchange

Early work dating back to the 1980s hypothesized that electron transfer occurs between aqueous Fe^2+^ and Fe­(III) (oxyhydr)­oxides,^31–34^ but direct experimental proof remained elusive. In 2004, Williams and Scherer^19^ conducted a key experiment providing definitive proof of electron transfer between aqueous Fe^2+^ and Fe­(III) in goethite. Their approach leveraged the selectivity of MBS for ^57^Fe atoms while ^56^Fe remained MBS-invisible. They added aqueous ^57^Fe^2+^ to goethite synthesized from pure ^56^Fe, and subsequently collected a Mössbauer spectrum of the solids (Figure 1A). Their results showed aqueous ^57^Fe^2+^ was oxidized and incorporated into goethite (Figure 2A,B), effectively creating a new layer of MBS-visible goethite.

**Figure 2:**
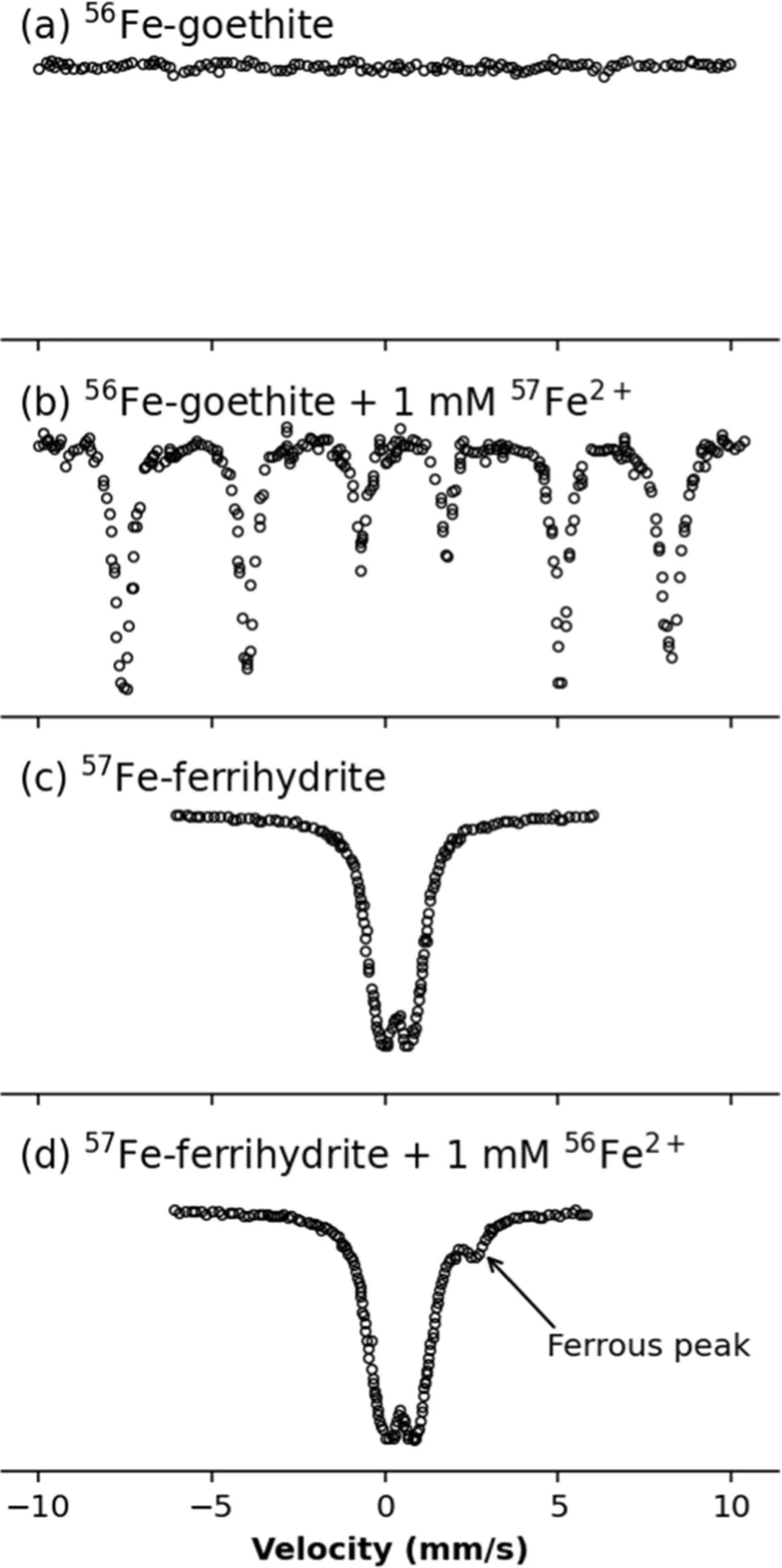
Mössbauer spectrum of ^56^Fe-goethite (a) before and (b) after reaction with aqueous^57^Fe^2+^, collected at 295 and 4.2 K, respectively. Mössbauer spectrum of ^57^Fe-ferrihydrite (c) before and (d) after reaction with ^56^Fe^2+^, collected at 140 K. Data extracted from Williams and Scherer 2004 ^19^ and digitized with PlotDigitizer.

As for the fate of the electron injected into the mineral, a reverse experiment provided the initial clues (Figure 1B). ^57^Fe-ferrihydrite was reacted with MBS-invisible aqueous ^56^Fe^2+^, and the Mössbauer spectrum of the reacted solids revealed an Fe­(II) doublet from ^57^Fe atoms initially in the mineral (Figure 2C,D). Combining these spectroscopic observations with aqueous-phase isotopic data revealed that electron transfer triggers structural atom exchange. This isotopic mixing was first demonstrated by Pedersen et al.,^35^ mostly for ferrihydrite and lepidocrocite, and was later shown by Handler et al.^36,37^ to extend to goethite, establishing that Fe atoms cycle between the aqueous and mineral phases.

Complementary to MBS-based studies, Yanina and Rosso^38^ demonstrated that a chemically induced potential gradient across hematite crystals resulted in the coupled growth on (001) basal surfaces and dissolution of distal (012) edge faces. This experiment and subsequent computational modeling studies provided direct evidence of electron mobility via electron hopping within Fe (oxyhydr)­oxides, enabling charge transfer between separated surface sites.^39,40^

Combining these findings led to a revised framework for interactions between aqueous Fe^2+^ and Fe­(III) in minerals.^36^ Aqueous Fe^2+^ sorbs onto the mineral, electron transfer oxidizes the Fe atom and grows a new layer, while electrons conducted through the bulk facilitate reductive dissolution and mixing of atoms from aqueous and solid phase. Gorski and Scherer^41^ provided a comprehensive historical overview and proposed a revised conceptual model for aqueous Fe^2+^-goethite electron transfer. Building on their foundation, the present paper examines how the application of stable Fe isotopes and MBS has enabled the Fe biogeochemical community to further investigate the mechanisms governing electron transfer and atom exchange.

Most initial studies on atom exchange focused on goethite and hematite, crystalline minerals that undergo Fe^2+^-catalyzed atom exchange via electron transfer, bulk conduction, and reductive dissolution, without macroscopic dissolution of the parent minerals. These studies did not find changes in morphology upon recrystallization,^36^ but later, TEM images revealed shifts in nanogoethite dimensions,^42^ while atomic force microscopy showed hematite surface pyramids and dissolution pits.^38^ Therefore, for goethite and hematite, the ability to trace isotopes moving from the aqueous to the solid phase clarified the extent of exchange and recrystallization of the solids.^43^ There have been attempts to demonstrate whether exchange reoccurs in the exchanged layer or if there are burial pathways of recrystallization.^44^ Recent probe tomography showed that aqueous atoms permeate at least 4 nm into the bulk mineral, suggesting active burial pathway rather than exchange stalling at the surface.^45^

Experiments employing ^56^Fe-goethite reacted with aqueous ^57^Fe^2+^ (Figure 1A) were used to study the controls on electron transfer and atom exchange. The presence of organic compounds, including natural organic matter, extracellular polysaccharides, and cell materials, as well as mineral Al-substitution, and common anions (PO_4_^3–^, CO_3_^2–^, SiO_4_^4–^) did not prevent electron transfer; specifically, they did not prevent the formation of a new layer of Fe­(III) mineral from atoms initially in the aqueous phase.^46^ The only exception was a long-chain (39 C) phospholipid, which completely suppressed the formation of a new layer of goethite.

However, although Fe^2+^ to goethite electron transfer is difficult to fully inhibit, it can be partially suppressed. Via MBS, this is evidenced by the accumulation of an Fe­(II)-containing phase at the ^57^Fe-enriched new layer of goethite (Figure 3A), and more clearly, by a slowdown in Fe atom exchange between aqueous and mineral phases (Figure 3B).^46^ Solution conditions, such as the concentration of protons,^43^ anions,^46^ and Fe^2+^, along with mineral properties including crystallinity, particle size, surface area, and substitution^37,43^ can further influence the extent of atom exchange. Again, by using aqueous ^57^Fe^2+^ and MBS (Figure 1A), it was experimentally demonstrated that the lack of surface defects suppresses electron transfer.^47^ Later, sequential exposure of ^56^Fe-goethite to aqueous ^56^Fe^2+^ followed by aqueous ^57^Fe^2+^ (Figure 1C) demonstrated that healing defects via reaction with Fe^2+^ slows the driving forces for electron transfer.^22^

**Figure 3:**
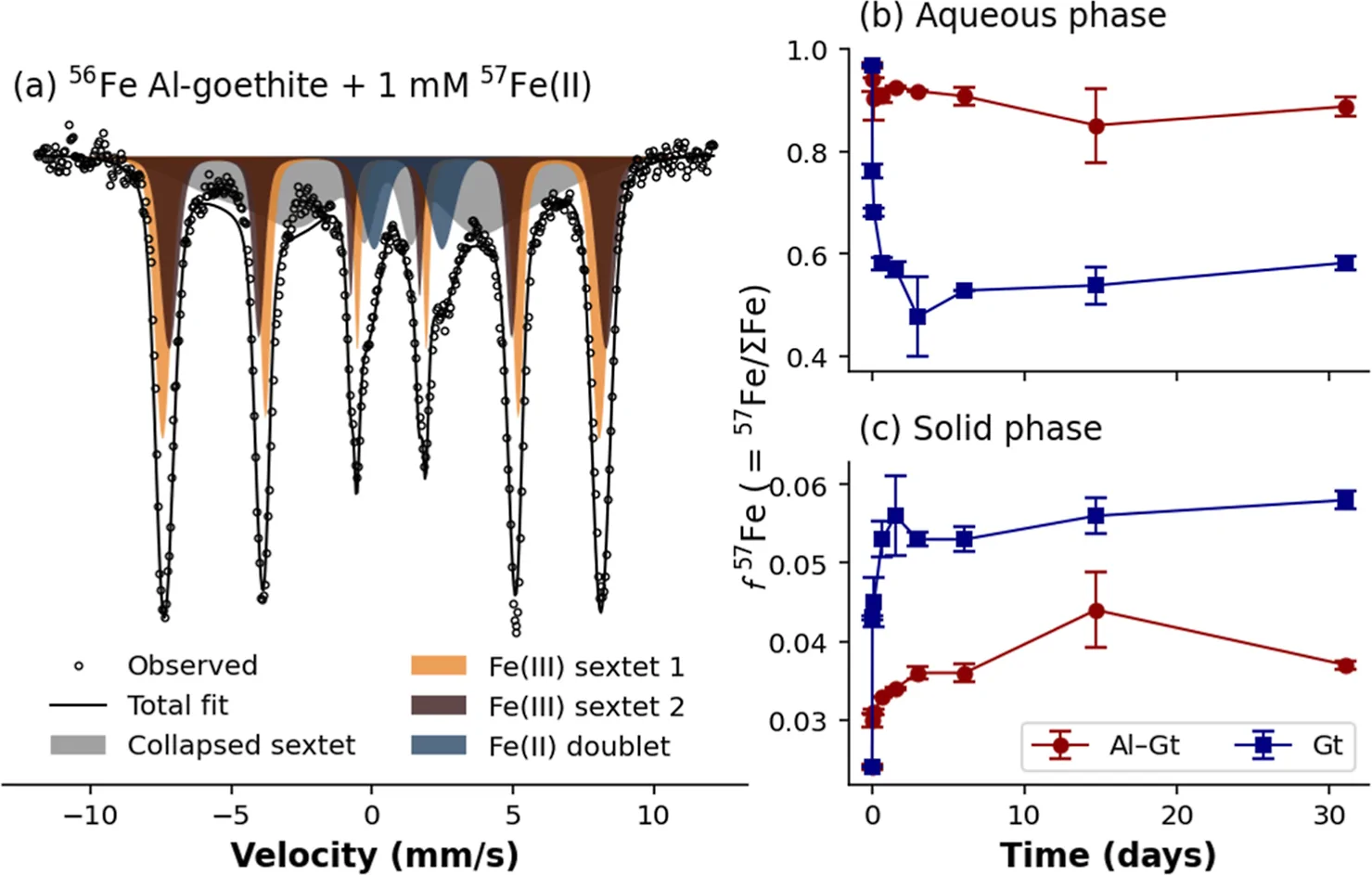
(a) Mössbauer spectrum of Al-substituted (9%) ^56^Fe-goethite reacted with 1 mM ^57^Fe­(II), collected at 77 K. (b) Fraction of ^57^Fe in the aqueous and (c) solid phases as ^56^Fe-goethite with and without Al-substitution react with ^57^Fe­(II). Data first published in Latta et al., 2012.^46^

Other minerals, such as magnetite, face a different fate when reacting with aqueous Fe^2+^. Although the net uptake of Fe^2+^ by magnetite seems to be limited by mineral stoichiometry, isotope tracking confirms extensive atom exchange between Fe atoms in the aqueous and mineral phase, with exchange values well beyond the amount of Fe found near the surface.^20,48^ MBS revealed that the atoms initially in the aqueous phase form a partially oxidized magnetite phase. While aqueous Fe^2+^ oxidation showed reduction of octahedral Fe^3+^ in the underlying magnetite,^20^ subsequent work suggested exchange occurs at both octahedral and tetrahedral sites.^48^

Thermodynamically unstable minerals, such as ferrihydrite, respond differently upon contact with aqueous Fe^2+^, undergoing transformation into more stable mineral phases.^10^ While some controls and products of ferrihydrite Fe^2+^-catalyzed transformation have been characterized by other techniques,^9,49^ the use of ^57^Fe isotopes and MBS has added specific insights into the mechanisms. For example, before ferrihydrite transforms into other phases, electron transfer and atom exchange between ferrihydrite and aqueous Fe^2+^ appear to occur first.^21^ Comprehensive tracing of Fe atoms in the aqueous and solid pool (Figure 1D) revealed that, during Fe^2+^-catalyzed transformation of ferrihydrite, Fe atoms initially in the aqueous phase predominantly formed lepidocrocite, whereas those originating from ferrihydrite primarily formed goethite,^24^ suggesting that Fe atoms from different pools play distinct roles. Targeted extraction procedures revealed the importance of the concentration of labile Fe­(III) species for nucleation on existing mineral surfaces,^50^ while ^57^Fe tracers revealed a signature similar to lepidocrocite lamellae.^51^ In mixed mineral systems, pre-existing goethite (Figure 1D) provides a template that promotes the Fe^2+^-catalyzed transformation of ferrihydrite into more goethite.^24^

Beyond ferrihydrite, combined isotopic and spectroscopic approaches have elucidated the mechanisms behind the transformation of other Fe-bearing phases. Lepidocrocite, for example, undergoes atom exchange without the formation of other mineral phases at low aqueous Fe^2+^ concentrations, but forms magnetite at higher aqueous Fe^2+^ concentrations.^52^ Similarly, jarosite and schwertmannite react with aqueous Fe^2+^ to form other Fe oxyhydroxides depending on the geochemical conditions. Atom exchange experiments revealed a reaction that was 1 order of magnitude slower in natural jarosite than in laboratory-synthesized samples.^53^ For schwertmannite, preadsorption of As­(III) inhibited the extent of transformation.^54^ Even Fe octahedrally coordinated within clay minerals undergoes electron transfer and atom exchange with aqueous Fe^2+^, demonstrating the dynamic nature of Fe in clay minerals.^55–57^

In summary, mineral slurry experiments with ^57^Fe tracers and MBS allowed in-depth atomic-level understanding of mechanisms and factors controlling Fe mineral transformations, allowing full control of porewater chemistry and mineral characteristics. However, in natural porous media systems multiple factors (and possibly mechanisms) influence transformation simultaneously, challenging the direct translation of laboratory findings into natural settings.

### Iron Mineral Transformation Processes in Soils

Understanding Fe-mineral transformation in soils requires approaches that capture the complexity and dynamic nature of natural systems. Several experimental approaches using natural-isotope-abundance Fe (^NA^Fe) minerals, including sequential extractions, diffusive gradients in thin films (DGT) and incubations of minerals inside mesh bags buried in soil,^58–61^ have demonstrated that the rates and products of transformation differ between soil-based experiments and controlled mineral-slurry experiments.^9,49,62^ As an example, spatial analysis of mineral distribution within ferrihydrite-filled mesh bags incubated in redox-active rice paddy soils under laboratory conditions confirmed that the formation of goethite and lepidocrocite dominated over magnetite^61^ despite bulk soil aqueous Fe^2+^ concentrations that favored magnetite formation in a mixed ferrihydrite suspension.^9^ The discrepancy between the transformation products in the presence or absence of soils, likely comes from limited Fe^2+^ diffusion into the mesh bag and the density of ferrihydrite sorption sites.^61^ This demonstrates that in soils, the distribution of zones with varying geochemical properties can define the outcomes of mineral transformation.

Since mineral transformation studies in soils demonstrate that reaction outcomes are dictated by the physical proximity of target minerals to other components of the incubation matrix, soil mineral transformation experiments should be investigated with a method that does not physically separate the target mineral phase from the soil environment. The use of ^57^Fe in combination with MBS provides such an approach, enabling selective tracking of introduced Fe within complex soil matrices. Its application in soil is enabled by the low natural abundance of ^57^Fe, which accounts for only 2.12% of total Fe atoms present in soils,^17^ where bulk soil Fe contents vary from <0.1 to >10%.^63^ The use of aqueous ^57^Fe as a tracer in soils has revealed rapid and dynamic Fe cycling between aqueous and solid Fe phases. Following the addition of aqueous Fe^2+^ to natural soils, isotopic analyses in the soil solution and MBS showed rapid adsorption of aqueous Fe^2+^ to reactive mineral surfaces and significant atom exchange with Fe oxyhydroxides over the course of 28–33 days;^25^ (Figure 1E). This atom exchange was initially dominated by short-range-ordered (SRO) minerals,^25,26^ but led to complete turnover of solid-phase Fe over time scales of years in the tropical soils studied.^26^ The extent and rate of these processes were further modulated by soil organic matter, which stabilized Fe oxyhydroxides and hindered transformation to more crystalline phases such as goethite and hematite.^25^

A complementary approach involves adding ^57^Fe-labeled synthetic minerals to soils (Figure 1F,G) to track the mineral transformation under natural conditions using MBS.^27^ Studies by Kretzschmar and co-workers using this approach across microcosm, mesocosm, and field settings confirmed that ferrihydrite transformation rates occur over weeks to months, depending on the soil,^27,30,64^ contrasting with expectations of hours to days in controlled laboratory studies.^9,49,62^ In contrast to mixed suspension experiments, transformation products in soils are dominated by poorly crystalline and thermodynamically unstable mineral phases, including green rust.^27,29,30^ Ferric phases represent a smaller fraction of the transformation products of ferrihydrite when incubated as a mixture with a soil matrix, with ferrous mineral phases being prevalent.^27,29,30^ These differences reflect the importance of dissolution-recrystallization processes under soil conditions, where transport mechanisms such as diffusion and advective flow further influence the spatial distribution of transformation products. Building on these findings, ^57^Fe labeling has also been applied to a range of other ferric mineral systems beyond ferrihydrite. These include the progressive transformation of lepidocrocite during cycles of flooding and drainage of rice paddy soils,^29^ the reactivity of jarosite and Al-substituted jarosite in acid sulfate paddy soils,^28^ and sulfidation-driven transformation of lepidocrocite and goethite in intertidal sediments.^65^

A key finding from this series of studies is that the mineral incubation method influences mineral transformation pathways. For example, Schulz et al.^29^ ran concurrent incubations of ferrihydrite in mesocosms using mesh bags containing either pure ^NA^Fe mineral or ^57^Fe-labeled mineral mixed with soil. The latter system better aligned with natural soils, allowing Fe minerals to be in close association with other organic and inorganic soil components. Results revealed diverging transformation processes (Figure 4). Physical separation of minerals from soil (e.g., in pure mineral mesh bag incubations) favors Fe^2+^-catalyzed mineral recrystallization or transformation. In contrast, direct mixing with soil components promotes microbially mediated Fe mineral dissolution over Fe^2+^-catalyzed mineral transformations. The predominance of Fe reduction ultimately drives precipitation of secondary ferrous or mixed-valent minerals, ranging from metastable phases like green rust to thermodynamically stable phases such as siderite, vivianite, and Fe sulfides.^27,64,65^ MBS was a key technique for first identifying green-rust in the environment,^66,67^ and by combining it with ^57^Fe has become a key technique for identifying the pathways of its formation and further transformation. Once formed, metastable phases are more long-lived in soils than in the absence of soils. The occurrence of ^57^Fe-labeled metastable phases among the products of transformation indicates that these phases may be more abundant in the environment than previously thought.

**Figure 4:**
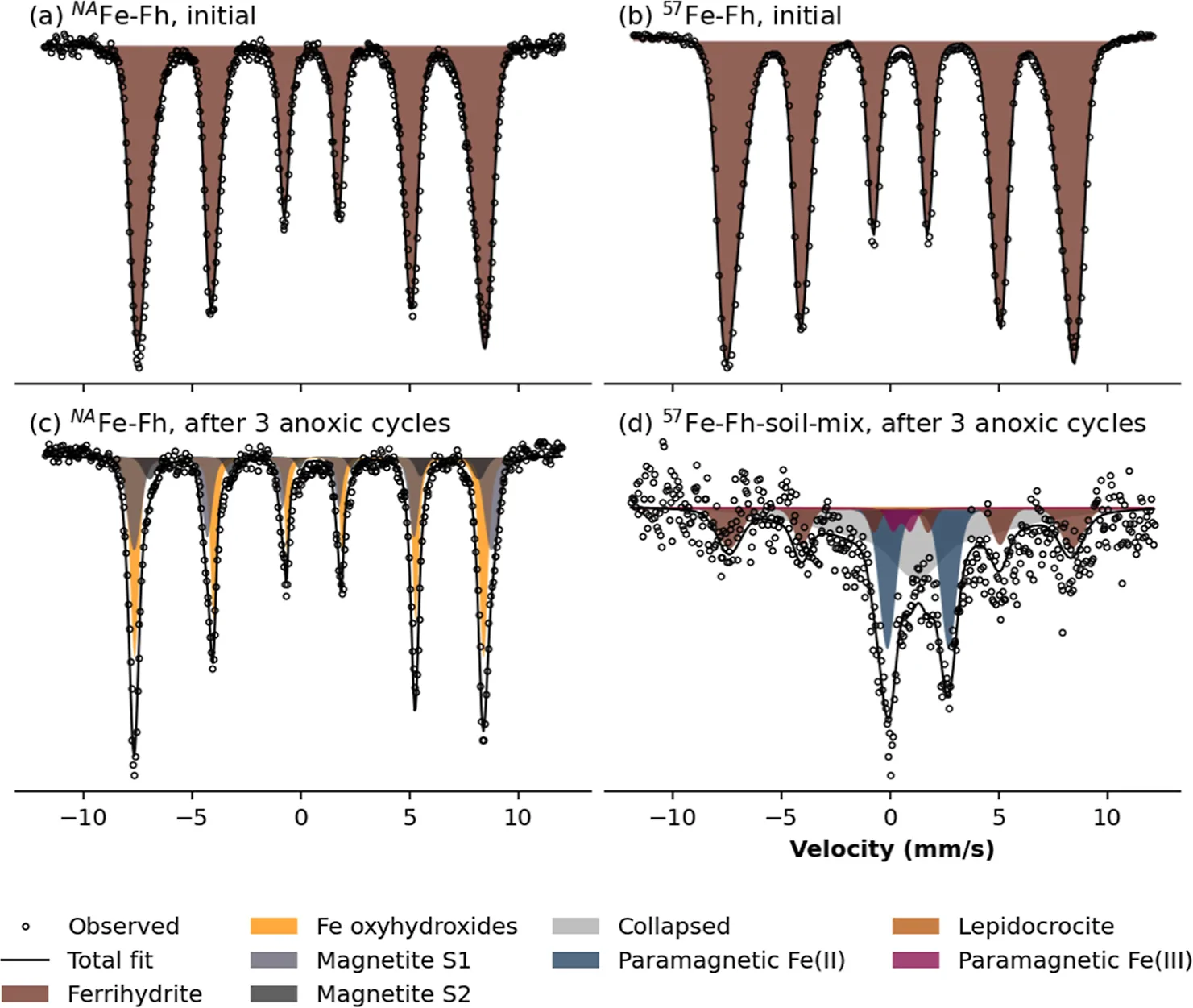
(a) Natural abundance ^NA^Fe-ferrihydrite and (b) isotopically labeled ^57^Fe-ferrihydrite before and (c,d) after concurrent incubation as pure minerals or mineral-soil mixes, respectively, in mesocosms that simulate redox fluctuations in a paddy soil environment. All data were collected at 5 K and first published in Schulz et al., 2023.^29^

Despite their widespread occurrence in anoxic soil horizons, sediments, and anoxic microsites, the stability, transformation and recrystallization of Fe­(II) minerals remain comparatively understudied, including by MBS.^18,68^ This gap is partly due to the inherent challenges of sampling and preserving oxygen-sensitive samples, but also reflects analytical limitations in their detection. In particular, complex and poorly constrained hyperfine parameters limit the identification and quantification of Fe­(II) minerals when they are not the dominant iron-bearing mineral phase. In MBS, many Fe­(II) mineral spectra magnetically order to octets, overlap with Fe­(II) present in clay minerals, and may exhibit wide ranges of hyperfine parameter values, making spectral fitting difficult. Careful choice of measurement temperature and improved fitting protocols are improving the identification of Fe­(II) minerals in soils and sediments.^18^ Looking ahead, integrated application of ^57^Fe as a tracer and MBS for detection holds great potential for resolving Fe­(II)-bearing mineral transformation pathways in complex environmental systems.

A combined view of the approaches for observing Fe mineral transformation processes in soils points to two main insights. First, the overall rate of Fe mineral transformation in mesh bags filled with pure minerals is constrained by diffusion and reactive transport of porewater compounds such as PO_4_^3–^, DOC, and Fe^2+^. Unlike natural soils, where colonization is more dispersed, pure mineral mesh bags get solutes from the outer soil. As reactive porewater constituents diffuse through the mesh bag, they sorb onto the initial mineral front, creating concentration gradients of those reactive compounds and microenvironments prone to different Fe mineral transformation pathways. Second, when minerals are in direct contact with the soil matrix, there are strong matrix effects, in which the classic geochemical factors that control Fe mineral transformation such as pH, Fe^2+^ concentration and sometimes even the characteristics of the solid phase, are modified by, for instance, competing reactions that consume the same reactants, and by the sorption of associated elements and other natural ligands to reactive mineral surfaces. Together, these insights suggest that without direct contact with soils, persistent laboratory-to-field gaps remain, compromising the application of laboratory-derived rates of Fe mineral transformation in geochemical modeling. In this scenario, combining ^57^Fe isotopes and MBS offers great opportunities to observe detailed Fe mineral transformation while taking into account reactive transport and matrix effects uniquely provided by close association with other soil components in nonmixed porous media.

### Influence of Mineral Transformations on Other Element Cycles

Although MBS provides information specifically on Fe, the combination of Fe isotopes and MBS has simultaneously provided insights into cycles of other elements that associate with Fe-bearing phases. The transformations of isotopically labeled Fe minerals in aqueous or soil matrices (Figure 1A,G) has revealed how structural substitution of Fe by other cations, such as Al or transition metals, affects Fe mineral stability and reactivity. Additionally, this approach has extended to the study of elements that bind with Fe during mineral formation, including S, P, C, and Si in Fe sulfides, phosphates, carbonates, and silicates, respectively. Both anions and cations may also be associated with minerals via surface adsorption or structural incorporation, further linking their environmental fate to the dynamics of their Fe mineral hosts.

Substitution of Fe by other elements in minerals such as goethite, hematite, and jarosite play a key role in regulating their reactivity and simultaneously function as reservoirs of trace elements in environmental systems. For example, studies on Al-substitution demonstrated that it significantly slows atom exchange in Fe minerals including goethite (Figure 3)^46^ and jarosite.^28^ Further studies revealed trace element release is greatly inhibited when an insoluble element is cosubstituted, with the total release decreasing exponentially as cosubstitution increases.^69^ This suggests that naturally occurring Fe (oxyhydr)­oxides containing insoluble elements are less susceptible to Fe^2+^-catalyzed recrystallization and exhibit a greater retention of trace elements and contaminants than pure mineral phases. While mineral transformation might be inhibited, recent studies also revealed that reaction with aqueous Fe^2+^ can improve the extractability of Al and P from minerals,^70,71^ with potential applications to the mining sector. Furthermore, the influence of such substitutions in Fe minerals is context dependent. For example, Al-substitution slows transformation of jarosite when Fe^2+^-catalysis is the dominant mechanism of transformation, but has less effect when jarosite is mixed with soil and reductive dissolution becomes the dominant transformation mechanism.^28^

^57^Fe isotopic labeling also provided key insights into coupled Fe–P and Fe–S transformation pathways in environmental systems. In Fe–P systems, incubation of ^57^Fe-ferrihydrite with and without adsorbed phosphate shows that adsorbed phosphate alters secondary minerals, shifting products from siderite and green rust toward vivianite.^30^ Importantly, vivianite formation would likely remain undetected in bulk soil analyses, despite its role as a key P-bearing phase driving P retention, highlighting the ability of Fe isotopic tracing to resolve hidden mineral products and link Fe and P cycling mechanistically.^30^ This isotopic approach is equally transformative when applied to sulfidic environments, where the interplay between Fe and S shape mineral transformations. Incubation of ^57^Fe-labeled goethite, lepidocrocite, and mackinawite in coastal soils revealed sulfidation of Fe­(III) phases and transformation of mackinawite into greigite,^65^ highlighting the tightly coupled Fe–S redox pathways that regulate mineral cycling in sulfate-reducing environments (Figure 5).

**Figure 5:**
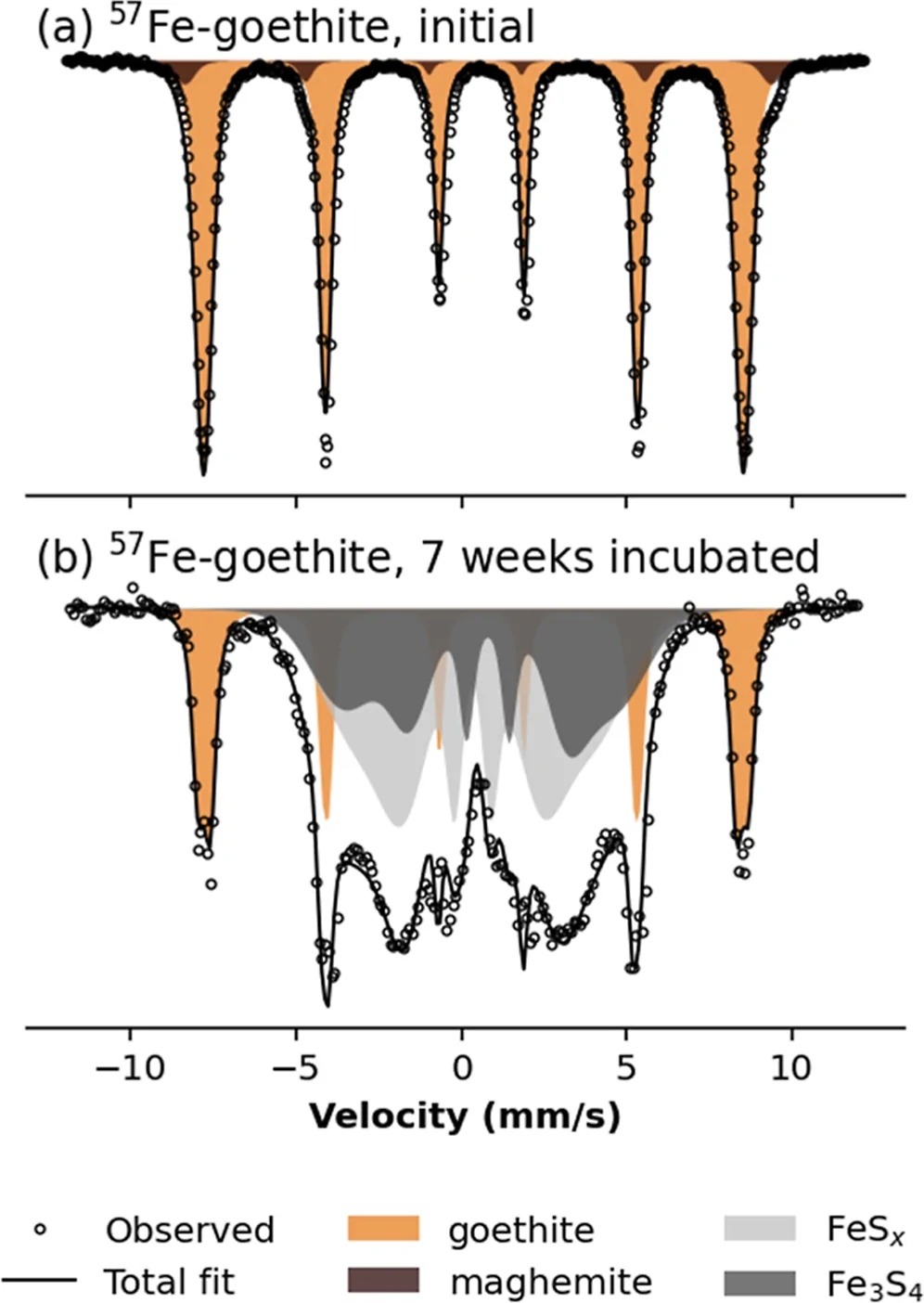
Mössbauer spectra of ^57^Fe-goethite mixed with intertidal sediments before and after 7 week incubation in a sulfidic site. Data were collected at 5 K and first published in Kubeneck et al., 2025.^65^

Coprecipitated or sorbed elements can alter Fe mineral reactivity and transformation pathways, often overriding behavior predicted from controlled laboratory systems that exclude foreign ions. This is evident across a range of experimental systems. For example, sorption of Si and coprecipitation of carbon with ferrihydrite can hinder atom exchange between aqueous and solid phases, and occasionally modify secondary minerals.^21,25^ Extending this to more complex environmental matrices, ThomasArrigo et al.^12,72^ coprecipitated ^57^Fe-ferrihydrite with ^13^C-labeled glucuronic acid and incubated the mixture in Icelandic soils (Figure 1F), showing that limited bioavailability of the organic component suppresses not only reductive dissolution of ferrihydrite but also the reduction of native soil Fe­(III) minerals. Similarly, coprecipitation of ferrihydrite with zinc in reducing sediments (Figure 1F) leads to rapid transformation into green-rust like phases, accompanied by release of Zn into the porewater to form secondary porewater species with local ligands such as sulfide.^73^ Collectively, these studies demonstrate that the outcomes of mineral transformation are strongly dependent on the ionic background of mineral transformations, and the conditions of mineral formation, pointing to a strong influence of complex environmental matrices on the outcomes of Fe mineral transformation processes.

## Implications

This perspective article highlights the various ways in which Fe isotopes are used to study Fe mineral transformation processes, from probing detailed mechanisms at the atomic scale to enabling precise measurements of in situ mineral transformation pathways in complex soil matrices. Initial applications of isotopically labeled Fe (oxyhydr)­oxides provided spectroscopic evidence of electron transfer between aqueous Fe^2+^ and Fe (oxyhydr)­oxides, followed by the recognition of Fe atom exchange between the aqueous and solid phases. Using isotopes, the permeation of Fe atoms into the bulk of minerals was demonstrated, and rates of atom exchange quantified. The approach has provided insights into both the mechanisms controlling electron transfer and the mobilization of trace metals during Fe mineral transformation, while simultaneously stabilizing the initial Fe mineral against transformation (Figure 6).

**Figure 6:**
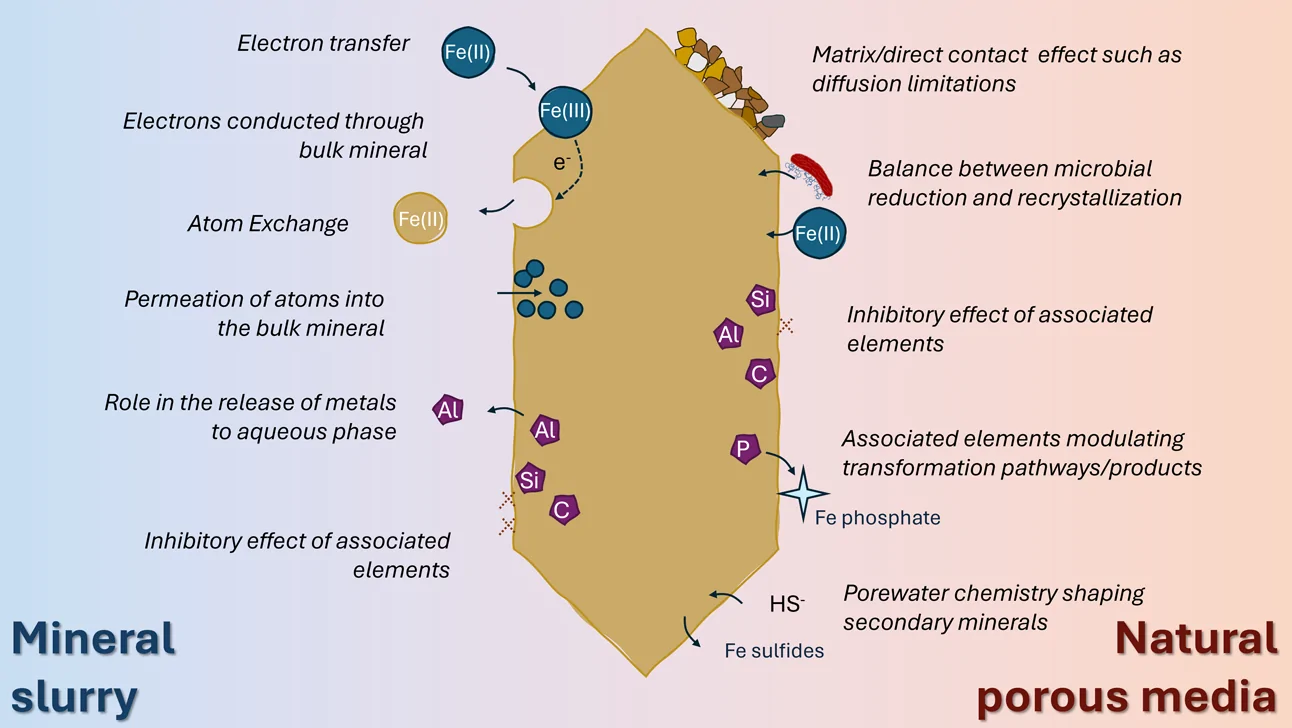
Mechanistic insights into iron cycling using stable isotope tracers and Mössbauer spectroscopy. While mineral slurries provide control necessary to isolate specific reaction pathways, experiments in natural porous media reveal how diffusion limitations and matrix effects influence iron mineral transformation.

Extending the application of ^57^Fe tracers to soils and sediments, either as aqueous spikes or as ^57^Fe-enriched minerals, has significantly advanced our understanding of Fe mineral transformation pathways in complex environmental matrices. In such systems, ^57^Fe isotopic tracing helps resolve transformation processes that are otherwise obscured due to its low natural abundance and interference from native Fe minerals. A key insight is that substituted, adsorbed, or coprecipitated elements, as well as neighboring crystal faces, can strongly modulate transformation pathways in soil. Similarly, incubations of ^57^Fe-labeled minerals within soil matrices reveal a shift toward microbially driven dissolution processes, followed by the formation of mixed-valent and reduced Fe phases that are less commonly observed in well-mixed laboratory systems (Figure 6). Ongoing development of experimental techniques, including advancement in collection and fitting routines for MBS, is enabling more detailed investigations of the effect of specific combinations of biogeochemical soil properties on the physical and mineralogical properties of Fe mineral transformation products.

While the use of ^57^Fe isotopes has offered numerous opportunities, it also comes with pitfalls, including the high cost of ^57^Fe, limited availability of MBS time, challenges in the interpretation of MBS results, and the diffusion of reduced ^57^Fe to the aqueous phase in open-field and mesocosm experiments. Therefore, its use must be intentional and linked to the importance of connecting molecular-scale reactions and macro-scale outcomes.

Looking forward, employing ^57^Fe-labeled Fe­(II) minerals opens new opportunities to resolve transformation pathways under dynamic redox conditions, including key sequences such as green rust oxidation and siderite formation. As applications of MBS to reduced Fe systems continue to expand, improvements in spectral interpretation will further strengthen its ability to resolve complex Fe­(II) mineral assemblages. Integrating isotopic tracing with time-resolved experiments could provide insights into Fe­(II)–Fe­(III) interconversion under transient oxic–anoxic conditions and to investigate Fe­(II)-mediated redox reactions, including Fenton-type processes relevant to organic compound degradation. Such methodological expansions will help close long-standing knowledge gaps and reframe how reduced Fe minerals are represented in biogeochemical redox models.

We further expect these techniques to expand beyond traditional Fe biogeochemistry into diverse engineering applications. The application can range from resolving the nuances of Fe redox reactions in remediation strategies to evaluating corrosion kinetics in infrastructure resilience. As iron remains central to both terrestrial biogeochemistry and man-made infrastructure, the future of the field lies in the synergistic advancement of these creative ways of using ^57^Fe as isotope tracers with traditional methodologies, and modeling approaches, each informing the other to close critical knowledge gaps.
